# Emergence of Indigenous Dengue Fever, Niger, October 2023

**DOI:** 10.3201/eid3007.240301

**Published:** 2024-07

**Authors:** Habibatou Idé Amadou, Saada Moussa, Ibrahim Issa Arzika, Hadiza Ousmane, Soumana Amadou, Balki Aoula, Abdoulaye Ousmane, Ibrahim Maman Laminou, Adamou Lagare

**Affiliations:** Centre de Recherche Medicale et Sanitaire, Niamey, Niger (H. Idé Amadou, I.I. Arzika, H. Ousmane, S. Amadou, B. Aoula, I.M. Laminou, A. Lagare);; Hopital National de Niamey, Niger (S. Moussa);; Universite Dan Dicko Dankoulodo, Maradi, Niger (A. Ousmane)

**Keywords:** Dengue, dengue fever, dengue virus, indigenous, Niger, viruses, vector-borne infections, arboviruses

## Abstract

Dengue fever is a growing worldwide public health concern. In mid-October 2023, multiple cases of uncommon febrile illness were reported among patients in Niamey, Niger. Fifteen samples were tested by using molecular methods, from which 7 (46.66%) were confirmed positive for mosquitoborne dengue virus belonging to serotypes 1 and 3.

Dengue fever is a mosquitoborne arbovirus infection, mainly reported in tropical and subtropical regions. Dengue fever is caused by the 4 types of dengue virus (DENV), 1–4 (*1*). Patients with DENV infection have onset of high and abrupt fevers tat are often accompanied by redness of the face, cutaneous erythema, myalgia, arthralgia, and headaches (*2*,*3*). In severe cases, healthcare workers will find evidence of hemorrhagic manifestations and signs of shock. The most common laboratory findings from a complete blood count are leukopenia, thrombocytopenia, and increased hematocrit (hemoconcentration) (*4*).

In recent years, DENV infection has progressed worldwide and become a major public health concern (*5*). Annually, >390 million infections are reported across the globe, of which 96 million have clinical manifestations and >25,152 result in death (*6**,**7*). DENV is now endemic in >34 countries in Africa (*7*). In 2023, a total of 171,991 suspected cases of dengue fever, including 70,223 confirmed cases and 753 deaths, were reported from 15 countries in West Africa. Burkina Faso is the most affected by dengue fever, accounting for 85% of reported cases and 91% of recorded fatalities (*8*). In Niger, there was a lack of data related to DENV infection until the recent confirmation of an imported case in November 2022 (*9*). In this report, we describe findings from 7 indigenous confirmed DENV cases in Niger. The Niger National Ethical Committee at the Ministry of Health approved the surveillance protocol as minimal risk research, and written consent forms were not required. Oral consent was obtained from the patients. All methods, including the use of human samples, were conducted in accordance with the Declaration of Helsinki.

During October 25–27, 2023, several public and private hospitals in Niamey reported cases of febrile syndrome including fever (>38°C), persistence of headaches despite administration of analgesics, muscle pain, and vomiting (Figure). None of the patient complaints included a body rash or hemorrhage, and the initial provider assessment was otherwise unremarkable. We conducted microscopic blood smear examinations of 15 patient samples; all were negative for malarial parasites. Our clinical management of the patients (hospitalized and ambulatory) consisted of symptom treatment. We observed thrombocytopenia and leukopenia an average of 72 hours after the initial examination. Of note, we tested all 15 patients for DENV infection within 7 days of symptom onset. 

**Figure:**
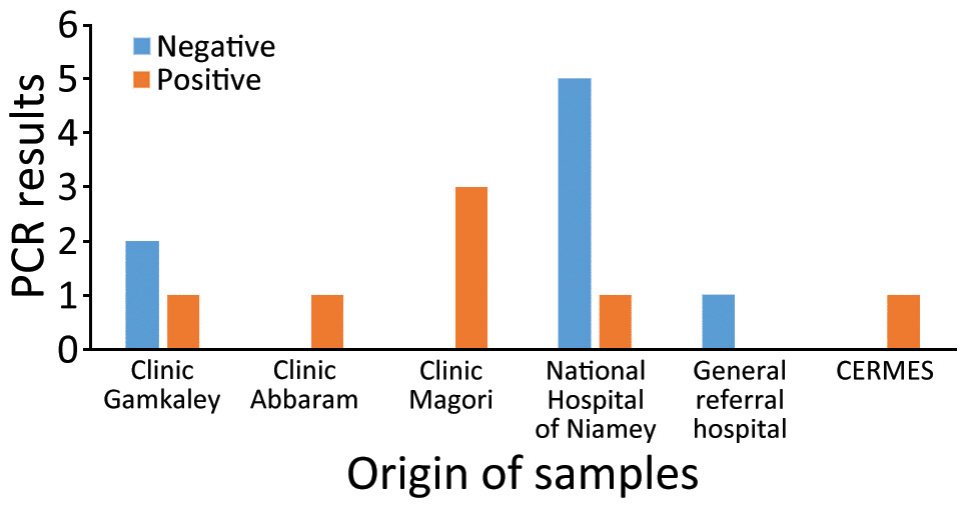
Distribution and results of dengue fever testing of suspected cases according to hospital or clinical origin in Niamey, Niger, October 2023. CERMES, Centre de Recherche Medicale et Sanitaire.

Because of the suggestive symptomatology of our cases and the ongoing DENV epidemic in neighboring countries, particularly Burkina Faso, we collected blood samples and sent them to the National Reference Laboratory for arboviruses at the Centre de Recherche Medicale et Sanitaire for virological confirmation. Testing was conducted by using qRT-PCR with specific primers and probes for the detection of the 3 main arboviruses, DENV, chikungunya, and Zika virus (*10*). Differentiation of DENV serotypes 1, 2, 3, and 4 was conducted by using the Dengue Real-TMGenotype kit (Sacace Biotechnology, https://sacace.com).

A total of 15 samples were tested for all 3 viruses, of which 7 (46.66%) were positive for DENV. No detection of chikungunya or Zika virus was confirmed. Among the patients tested, 8 (53%) were male and 7 (47%) female; mean age was 34 (range 13–76) years. In the confirmed cases of DENV, the average age was 36 (range 13–51) years, 4 (57%) were male, and 3 (43%) were female (Table). The 7 confirmed DENV cases were linked to residents from Niamey, the capital city of Niger, and had no reported travel history outside the county. The detection of DENV serotypes was successful in 4 of the positive samples; 2 were DENV-1 and 2 DENV-3. Serotyping was not possible for the other 3 samples because of low viral levels (Table). The 7 cases, both hospitalized and ambulatory, recovered from the DENV infection without any severe complications.

**Table:** Clinical and paraclinical characteristics of indigenous dengue confirmed patients in Niamey, Niger, October 2023*

Variables	Case 1	Case 2	Case 3	Case 4	Case 5	Case 6	Case 7
Sex	M	M	F	F	M	F	M
Age, y	40	13	47	28	51	45	16
Signs/symptoms	Fever, headache, muscle pain	Fever, headache, muscle pain	Fever, headache, muscle pain	Fever, headache, muscle pain, vomiting, vertigo	Fever, headache, muscle pain, vomiting, asthenia	Fever, headache, muscle pain	Fever, muscle pain
Mode of care	Hospitalized	Hospitalized	Hospitalized	Ambulatory	Hospitalized	Hospitalized	Hospitalized
Fever onset date	2023 Oct 10	2023 Oct 19	2023 Oct 22	2023 Oct 21	2023 Oct 20	2023 Oct 23	2023 Oct 20
Sampling date	2023 Oct 25	2023 Oct 25	2023 Oct 25	2023 Oct 27	2023 Oct 27	2023 Oct 27	2023 Oct 27
Dengue typing	Positive	Positive	Positive	Positive	Positive	Positive	Positive
Dengue serotyping	DENV-3	DENV-3	ND	DENV-1	ND	ND	DENV-1

*DENV, dengue virus; ND, serotyping not done because of low viral load.

After the official notification to the National Health Authorities, public health actions were implemented to contain the spread of the virus. An investigation team was dispatched by the Ministry of Health to investigate all confirmed cases of dengue fever. Prevention and control measures were put into place, namely awareness raising at the community level and awareness raising and training of healthcare personnel on the diagnosis and management of dengue fever. An entomologic survey was also conducted around patients’ residences and hospitalization facilities, but 2 *Aedes* spp. mosquitoes captured and tested yielded no positive results for dengue, chikungunya, or Zika viruses.

In conclusion, we describe 7 indigenous cases of dengue fever in Niger. Dengue fever cases are underreported in Africa, where it is often misdiagnosed as malaria (*1*). Misdiagnosis and underreporting highlights the need to train healthcare staff on the recognition and diagnosis of dengue fever. Strong vector control measures are also beneficial for containing the spread of dengue fever (*4*).
